# Venous-Left Atrial Extracorporeal Membrane Oxygenation Configuration Use in Right Ventricular Failure and Tricuspid Ring

**DOI:** 10.1016/j.jaccas.2022.07.020

**Published:** 2022-10-05

**Authors:** Harish Ravipati, Mouhamed Amr Sabouni, Tejasri Kodavaluru, Hassan Alkhawam, Mustafa I. Ahmed

**Affiliations:** a Section of Advanced Heart Failure, Department of Medicine, University of Alabama at Birmingham, Birmingham, Alabama; b Division of Interventional and Structural Cardiology, Department of Medicine, University of Alabama at Birmingham, Birmingham, Alabama; c Gandhi Medical College, Secunderabad, India

**Keywords:** cardiac assist devices, right ventricle, tricuspid valve, AS, aortic stenosis, LVEF, left ventricular ejection fraction, MR, mitral regurgitation, PA, pulmonary artery, RA, right atrial, RV, right ventricular, RVAD, right ventricular assistive device, TEE, transesophageal echocardiogram, TH, tandem heart, TR, tricuspid regurgitation, TV, tricuspid valve, VA ECMO, veno-arterial extracorporeal membrane oxygenation

## Abstract

Mechanical circulatory support devices are used to support the heart in cardiogenic shock. We present a case of demonstrating the feasible use of left ventricular assistive device with reverse configuration to support severe right ventricular failure in a patient with recent tricuspid annuloplasty ring.

## Central Illustration

**Figure:**
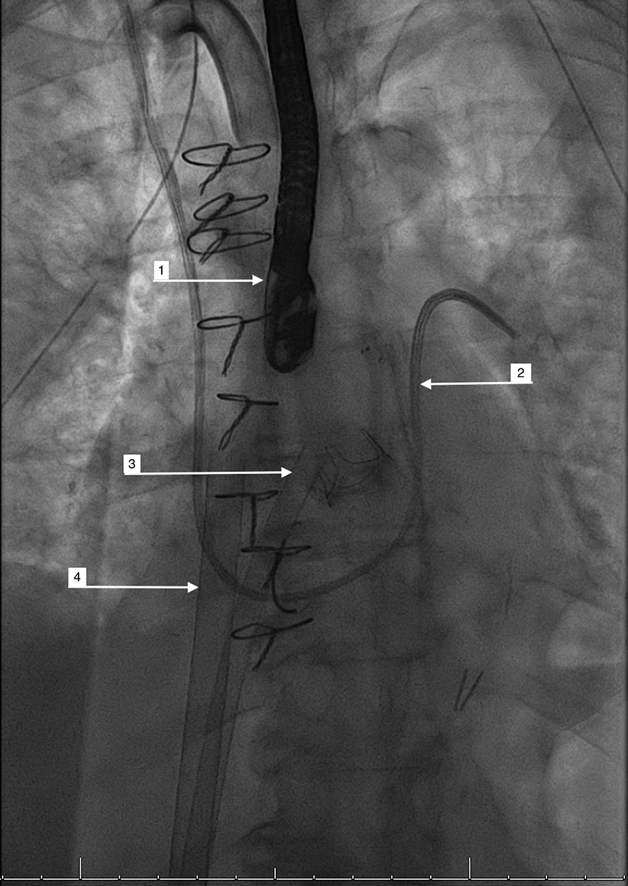

## History of Presentation

Our patient is a 78-year-old Caucasian female who presented to an outside hospital with symptoms of heart failure. She was found to have severe rheumatic mitral regurgitation (MR), severe aortic stenosis (AS), and moderate tricuspid regurgitation (TR), so she was referred to our facility for a surgical evaluation regarding her valvular heart disease. On admission, she had severe limitation of physical activity and shortness of breath with mild exertion, and symptoms were consistent with New York Heart Association functional class III. Initial transthoracic echocardiogram showed left ventricular ejection fraction (LVEF) of 35% based on Simpson’s method and right ventricular (RV) function was moderately reduced (Video 1). She was treated with intravenous diuretics for acute heart failure exacerbation to optimize her medically before valve surgery. Right heart catheterization (RHC) was performed on admission and right atrial (RA) pressure was 18 mm Hg, RV pressure was 25/9 mm Hg with mean pressure of 14 mm Hg, pulmonary artery (PA) pressure was 26/16 with mean pressure of 10 mm Hg, and pulmonary capillary wedge pressure was 11 mm Hg. A transesophageal echocardiogram (TEE) showed severe TR with RV base as 5.3 cm, tricuspid valve (TV) annulus was 4.7 cm, tricuspid annular plane systolic excursion was 0.6 cm, moderate MR with annuloplasty ring with effective mitral regurgitant orifice was 0.28 cm/s^2^, vena contracta area was 0.26 cm/s^2^, severe AS with aortic valve area was 0.82 cm^2^, and RV function was moderately to severely reduced. After a period of optimization and heart team discussion, cardiac surgery was chosen because of mixed MR, AS valve disease, and TR; the percutaneous option was not considered.^1^ She underwent tricuspid annuloplasty with 26-mm Medtronic Tri-Ad band, mitral valve replacement with 27-mm St. Jude epic tissue valve, and aortic valve replacement with 19-mm Edwards Inspiris tissue valve. She was not able to be weaned off of the ventilator during the immediate postoperative period and she was extubated later to bilevel positive airway pressure. She was admitted to the cardiac intensive care unit and continues to have biventricular failure on repeat TEE and was on phenylephrine and epinephrine infusions.

**Learning Objectives**

• To manage the RV failure and cardiogenic shock in patients with tricuspid annuloplasty ring with venous-left atrial ECMO configuration.
• To discuss the use of mechanical circulatory support devices in cardiogenic shock.

## Medical History

The patient had heart failure with reduced ejection fraction, recent coronary artery disease status after right coronary artery stenting 3 months before presentation, remote history of MR after robotic mitral valve 9 years ago, carotid artery disease, atrial flutter post-ablation, hypertension, and scleroderma.

## Differential Diagnosis

Initial presentation with shortness of breath and functional limitation was due to acute heart failure with reduced ejection fraction and lung pathology was low on the differentials. Shock in the postoperative period was more likely from biventricular heart failure leading to cardiogenic shock and other differentials include septic shock, worsening pulmonary hypertension, and presence of an obstructive component like pulmonary embolism.

## Investigations

Admission laboratory values were significant for elevated creatinine of 1.6, glomerular filtration rate of 31 mL/min/1.73 m^2^, hemoglobin of 10.6 g/dL, and brain natriuretic peptide of 733 pg/mL. Blood cultures were done to investigate the infectious etiology after the patient was diagnosed with shock after valve surgery. Blood cultures were positive for nonresistant *Pseudomonas aeruginosa*. Computed tomography of the chest, abdomen, and pelvis with contrast to rule out the source of the pseudomonas bacteremia, which revealed bilateral lung consolidations and pulmonary embolism, was not noted.

## Management

**Figure 1:**
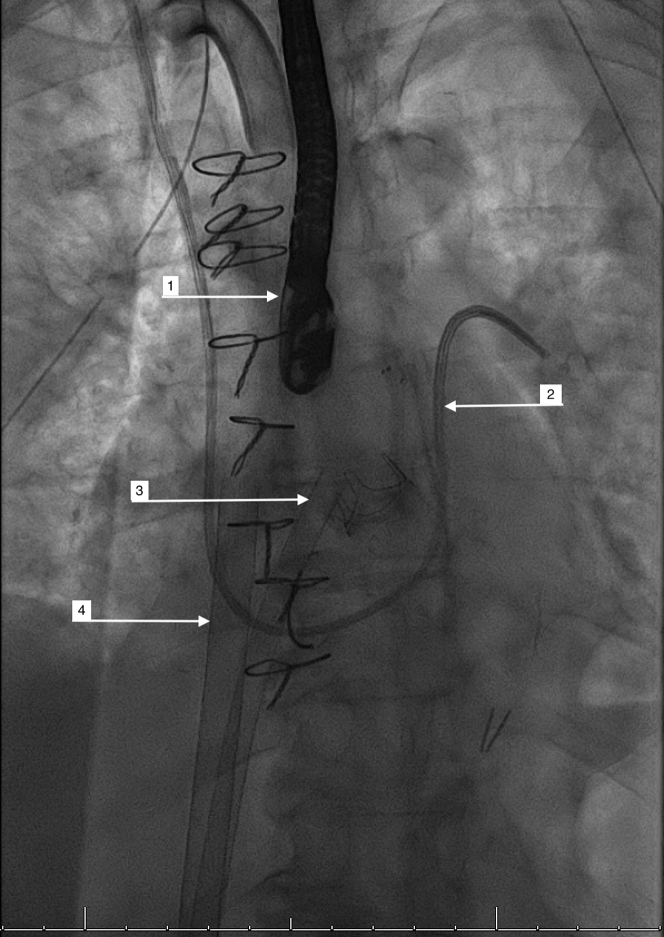
Fluoroscopic Image of Venous-Left Atrial Extracorporeal Membrane Oxygenation configuration Cannula Placement **Arrows** represent (1) transesophageal echocardiography probe, (2) Swan-Ganz catheter, (3) left atrial cannula, and (4) venous cannula.

Hospital course was complicated by worsening acute kidney injury requiring continuous renal replacement therapy with improved creatinine. She received Cefepime for Pseudomonas bacteremia and repeat blood and urine cultures showed resolution of infection. Because of worsening hypotension after successful treatment of pseudomonas infection, repeat TEE was done and it showed worsening of biventricular dysfunction and she was started on Milrinone and inhaled nitric oxide. Because the oxygen requirements continued to increase, she was reintubated and bronchoscopy was done, which did not reveal any significant findings. Her hemodynamic status continued to worsen with LVEF of 25% and severe RV failure on repeat TEE; norepinephrine and vasopressin infusions were added. Despite all the vasopressor support, she continued to have worsened cardiogenic shock and she was cannulated with veno-arterial extracorporeal mechanical oxygenation (VA ECMO) with intravenous heparin infusion. Repeat right-sided heart catheterization showed RA pressure of 12 mm Hg, RV pressure of 30/10 mm Hg with mean pressure of 11 mm Hg, and pulmonary wedge of 12 mm Hg on VA ECMO 4 L/min. Because she was still intubated and no improvement was noted in the respiratory status, a tracheostomy was done and a percutaneous gastrostomy tube was placed. After nearly 2 weeks of VA ECMO support, repeat TEE showed improved LVEF to 45% and severely worsened RV function (Videos 2 and 3). Because she was ventilator dependent with severe RV failure and improved LVEF, the plan was to reconfigure VA ECMO. A decision was made to wean her off of VA ECMO and insert Protek Duo which is a single right Internal Jugular access centrifugal flow right ventricular assistive device (RVAD) to pass through the TV. But, as she had recent tricuspid annuloplasty and ring across the TV, single right internal jugular access centrifugal flow RVAD was unable to be inserted and the decision was to proceed with reverse tandem heart which is venous to left atrial (LA) ECMO configuration (Figure 1). The right femoral vein access was used as the draining cannula of the venous to LA ECMO circuit and the left femoral vein was accessed placing the LA infusion cannula after trans-septal puncture. So now we had a draining cannula in the right femoral vein and, after oxygenating the blood, the return cannula in the left atrium. This venous to LA ECMO configuration was used to support the failing right ventricle in the setting of the new annuloplasty ring and improved LVEF. After reverse configuration with venous to LA ECMO placement, from day 1 to day 2 there was an improvement noted in central venous pressure from 18 mm Hg-12 mm Hg, PA pulsatility index from 0.83-1.33, and systemic vascular resistance/systemic vascular resistance index improved from 1,635/268 dynes/s/cm^5^ to 1,393/221 dynes/s/cm^5^. Although venous to LA ECMO configuration improved RV failure, the patient died due to multiorgan failure and septic shock in the next few days.

## Discussion

To our knowledge this is the first case reported to have a venous to LA ECMO configuration placement, an acute mechanical circulatory support with venous to left atrial configuration in a patient with tricuspid annuloplasty and severe RV failure. Acute mechanical circulatory support devices are indicated in cardiogenic shock in patients with either left ventricular failure or RV failure or biventricular failure. Common left ventricular assist devices (LVAD) include intra-aortic balloon pump, transcutaneous LVAD 2.5, transcutaneous LVAD 5, VA ECMO, and LA to femoral artery ECMO configuration. LA to femoral artery ECMO configuration improves cardiac output by 3.5 L/min, which is in between transcutaneous LVAD 2.5 and 5. RVADs include single femoral venous access axial flow RVAD, dual femoral venous access centrifugal flow RVAD, and single right internal jugular access centrifugal flow RVAD.^2^ VA ECMO is used for cardiogenic shock and respiratory support; venovenous ECMO is used for respiratory failure without cardiogenic shock. The patient was initially placed on VA ECMO due to cardiogenic shock from biventricular failure along with respiratory failure. Later in the course, because she was a candidate for RVAD because of isolated severe RV failure, VA ECMO was decannulated. Single right Internal Jugular access centrifugal flow and dual femoral venous access centrifugal flow RVAD remove blood for oxygenation from the RA and deliver it to the PA with a separate catheter to bypass RV, whereas single femoral venous access axial flow RVAD delivers the blood to the PA from the RA without oxygenation.^3^ Dual femoral venous access centrifugal flow RVAD and single right Internal Jugular access centrifugal flow RVAD decrease RA pressure and increase mean PA pressure.^3^ Because single right Internal Jugular access centrifugal flow, single femoral access axial flow RVAD and dual femoral venous access centrifugal flow RVAD need to pass the TV and she had a recent tricuspid annuloplasty with a tricuspid ring, these support devices were difficult to pass through the TV. Transcutaneous LVAD 2.5 and 5 were not considered because they directly off load left ventricle and to use as RVAD with reverse configuration they need to be passed through the TV, which is difficult in this case because of TV annuloplasty, and they are also designed to access the arterial system, whereas LA to femoral artery configuration LVAD and dual femoral venous access centrifugal flow RVAD were designed to access the venous system. To decrease the preload on the RV, the only available mechanical supportive device is to use reverse LA to femoral artery ECMO configuration which is venous to LA ECMO configuration. Its main objective is to oxygenate the blood obtained through the femoral vein and deliver it to the left atrium to bypass RV. Studies have shown that hospital mortality rate is between 44% and 57% from dual femoral venous access centrifugal flow RVAD.^3^ Although the mortality rate is high with these devices, advancements in the field of structural cardiology improve care in patients with cardiogenic shock. This case provides insights on the indications of using venous to LA ECMO configuration.

## Follow-up

Despite aggressive measures with acute mechanical circulatory support by VA ECMO and venous to LA ECMO configuration, the patient’s hemodynamic status continued to deteriorate, and the decision was made to withdraw care.

## Conclusions

Venous to LA ECMO configuration can be used as an acute mechanical circulatory support in patients who have severe TV stenosis or tricuspid annuloplasty ring with severe isolated RV dysfunction.

## Funding Support and Author Disclosures

The authors have reported that they have no relationships relevant to the contents of this paper to disclose.

## Appendix

Supplemental Video 1 Four-chamber view of transthoracic echocardiogram showing decreased left ventricular function on admission.

Supplemental Video 2 Transesophageal echocardiogram performed 1 day before reverse tandem heart placement showing improved left ventricular function from apical view.

Supplemental Video 3 Transesophageal echocardiogram performed 1 day before reverse tandem heart placement showing improved left ventricular function.
